# Copy number variations of the extensively amplified Y-linked genes, *HSFY* and *ZNF280BY*, in cattle and their association with male reproductive traits in Holstein bulls

**DOI:** 10.1186/1471-2164-15-113

**Published:** 2014-02-08

**Authors:** Xiang-Peng Yue, Chad Dechow, Ti-Cheng Chang, James Melton DeJarnette, Clifton Eugene Marshall, Chu-Zhao Lei, Wan-Sheng Liu

**Affiliations:** 1 College of Animal Science and Technology, Northwest A&F University, Yangling, Shaanxi 712100, China; 2 Department of Animal Science, The Center for Reproductive Biology and Health (CRBH), College of Agricultural Sciences, The Pennsylvania State University, University Park, Pennsylvania, PA 16802, USA; 3 Select Sires Inc, Plain City, OH 43064, USA; 4 Current address: Department of Plant Pathology, University of California Davis, Davis 95616, USA

**Keywords:** CNVs, *HSFY*, *ZNF280BY*, Male fertility, Testis size, Sire conception rate, Cattle

## Abstract

**Background:**

Recent transcriptomic analysis of the bovine Y chromosome revealed at least six multi-copy protein coding gene families, including *TSPY, HSFY* and *ZNF280BY*, on the male-specific region (MSY). Previous studies indicated that the copy number variations (CNVs) of the human and bovine *TSPY* were associated with male fertility in men and cattle. However, the relationship between CNVs of the bovine Y-linked *HSFY* and *ZNF280BY* gene families and bull fertility has not been investigated.

**Results:**

We investigated the copy number (CN) of the bovine *HSFY* and *ZNF280BY* in a total of 460 bulls from 15 breeds using a quantitative PCR approach. We observed CNVs for both gene families within and between cattle breeds. The median copy number (MCN) of *HSFY* among all bulls was 197, ranging from 21 to 308. The MCN of *ZNF280BY* was 236, varying from 28 to 380. Furthermore, bulls in the *Bos taurus* (BTA) lineage had a significantly higher MCN (202) of *HSFY* than bulls in the *Bos indicus* (BIN) lineage (178), while taurine bulls had a significantly lower MCN (231) of *ZNF280BY* than indicine bulls (284). In addition, the CN of *ZNF280BY* was positively correlated to that of *HSFY* on the BTAY. Association analysis revealed that the CNVs of both *HSFY* and *ZNF280BY* were correlated negatively with testis size, while positively with sire conception rate.

**Conclusion:**

The bovine *HSFY* and *ZNF280BY* gene families have extensively expanded on the Y chromosome during evolution. The CN of both gene families varies significantly among individuals and cattle breeds. These variations were associated with testis size and bull fertility in Holstein, suggesting that the CNVs of *HSFY* and *ZNF280BY* may serve as valuable makers for male fertility selection in cattle.

## Background

Recent progress on sequencing of the mammalian Y chromosomes has revealed that the amplification of the male-specific region on the Y (MSY) is a unique phenomenon during the mammalian sex chromosome evolution [1]– [3]. Because this amplification process was lineage-dependent, it resulted in different structures and contents of DNA sequences and genes in the MSY regions in different lineages, leading to different sizes of MSYs (and Y chromosomes) in mammals. The MSY was found to be enriched in multi-copy gene families; and these multicopy gene families, irrespective of the lineage in which they originated, are all expressed predominantly or solely in testis and are involved in spermatogenesis and male fertility [1]– [8]. Although the copy number variations (CNVs) of the Y-linked gene families have not been well-studied in mammals because of the unavailability of the Y chromosome sequences, a limited number of studies have demonstrated that Y-linked CNVs are associated with male fertility in humans [9,10] and cattle [11,12].

The gene content of MSY in the bovine (*Bos taurus*) Y chromosome (BTAY) has recently been studied [2], which is significantly different from that of the human MSY [1]. There are at least six multi-copy protein-coding gene families on BTAY, including *TSPY* (*testis-specific protein*, *Y-encoded*), *HSFY* (*heat-shock transcription factor*, *Y-linked*), *PRAMEY (preferentially expressed antigen in melanoma*, *Y-linked)*, *ZNF280AY* (*zinc finger protein 280A*, *Y-linked*), *ZNF280BY* (*zinc finger protein 280B*, *Y-linked*) and *EGLY* (*envelope glycoprotein like*, *Y-linked*) [2]. These gene families can be classified into two groups based on their evolutionary origins: proto-sex chromosome related genes, including *TSPY*, *HSFY*, and *EGLY*, of which *TSPY* and *HSFY* are conserved in some other mammalian species [13], and autosome-to-Y transposed genes, including *ZNF280AY*, *ZNF280BY*, and *PRAMEY*, which are bovid-specific [3,4]. Previous studies revealed that the bovine *TSPY*, *HSFY, ZNF280AY*, and *ZNF280BY* gene families were extensively amplified (up to 250 copies) on the Y during evolution [2,11,14]– [17], while the *PRAMEY* and *EGLY* gene families were less amplified (3-30 copies) [2,12]. Hamilton *et al.*[11] reported that the *TSPY* copy number (CN) was positively correlated with bull fertility and negatively correlated with the *TSPY* (mRNA) expression level in the testis. Yue *et al.*[12] has recently identified that the CNV of the bovine *PRAMEY* was negatively associated with testis size, non-return rate and percentage of normal sperm.

*HSFY* is a member of the *heat shock transcriptional factor* (*HSF*) family that is found in multiple copies on the Y chromosome and conserved in a number of species [17]. The HSFs regulate the expression of heat shock proteins (HSPs) through binding to the sequences located on the *HSP* genes, which are thought to have important roles in spermatogenesis [18]. The human *HSFY* is expressed predominantly in the testis [19]. Low or absent expression of *HSFY* in spermatogenic cells could lead to maturation arrest in men [18], and the deletion of *HSFY* on the human Y chromosome was associated with the unexplained cases of idiopathic male infertility [20]. In cattle, *HSFY* likely plays a similar role in spermatogenesis as it does in humans because the bovine *HSFY* is expressed specifically in the testis [2,17]. The CN of *HSFY* varies among species, for example, the human Y has 2 copies [1], while the feline Y possesses 8 copies [21]. A recent report found 70 copies of *HSFY* on BTAY with no CNV among 24 Holstein bulls [17]. However, another recent report found as many as 192 copies in a Hereford bull (L1 Domino 99375) that was used for the BTAY sequence project (GenBank acc. no. CM001061, Project ID: 20275) [2].

*ZNF280BY* is a newly identified BTAY-specific multi-copy gene family, which belongs to the family of zinc finger proteins [2,3]. Although zinc finger proteins are among the most abundant and functionally diverse proteins in mammalian genomes [22], little is known of the functions of *ZNF280BY* in mammals. In Drosophila, the ortholog of *ZNF280BY, i.e. suppressor of Hairy-wing* [*Su(Hw)*], encodes a protein that binds to an insulator element within a gypsy retrotransposon [23,24], implying that *ZNF280BY* may be involved in transcription regulation. The bovine *ZNF280BY* gene family was found to be expressed in different developmental stages of the testis with the sense RNA present in all cell types of seminiferous tubules while the antisense RNA present only in spermatids, signifying a role in spermatogenesis [3]. It was estimated that approximately 234 *ZNF280BY* genes are present on the Y chromosome of the Hereford bull, L1 Domino 99375 [2].

To date, there are few studies on the CNVs of either *HSFY* or *ZNF280BY* and their association with semen quality and male fertility traits in cattle. The objective of this study is to determine whether the bovine *HSFY* and *ZNF280BY* have any CNVs among individuals and breeds, and if their CNVs are associated with bull reproductive traits. Here, we provide evidence that the gene CN varies from 21 to 308 with a median of 197 for *HSFY,* while 28 to 380 with a median of 236 for *ZNF280BY* among cattle breeds. We found that the CNVs of both *HSFY* and *ZNF280BY* are associated with male reproductive performance.

## Methods

### Animal ethics statement

Animal care and seminal collection were carefully followed the Certified Semen Services (CSS) - Artificial Insemination Center (AIC) Animal Management Guidelines (Revised November 2011) (http://www.naab-css.org/about_css/practices2011.htm).

### Collection of samples and relevant information

A total of 460 bulls from 15 breeds were analyzed in this study (Table 1). Of these, 257 are Holstein bulls used in artificial insemination (AI), which have phenotypic records. Out of 257 Holstein bulls, 140 have records in scrotal circumference (SC), age adjusted relative scrotal circumference (RLSC), post thaw motility (PTM), incubated motility (IM), percentage of normal sperm (PNS) and percentage of intact acrosome (PIA). In addition, 82 (of the 140) bulls have data on sire conception rate (SCR), which is a bull fertility evaluation system recently developed by USDA (http://aipl.arsusda.gov/reference/arr-scr1.htm), and 102 (of the 140) bulls have data on relative breeding efficiency (RBE), which is an in-house bull fertility evaluation parameter estimated by Select Sires Inc. (Plain City, OH, USA). RBE uses a similar methodology to SCR but contains only a one-year rolling database [12]. The remaining 117 Holstein bulls have data on non-return rate (NRR). NRR is a traditional methodology to evaluate bull fertility that a cow was inseminated, and was not called to re-service within a given amount of time (usually 60 days) for the first service [12]. The remaining animals were either from the bovine HapMap Project (13 breeds) [25] or from a Chinese local cattle breed (Qinchuan), which do not have any phenotypic records and pedigree information. The Hereford bull L1 Domino 99375 (American Hereford Association registration number 41170496) was sequenced in the BTAY sequencing project (https://www.hgsc.bcm.edu/content/y-chromosome-genome-project) and was used as a calibrator for Y-linked gene copy number estimation [12]. In addition, paternal pedigree data from 192 Holstein bulls were available from the public databases. These animals were descendants of 4 patrilineal founders (HOUSA1427381, HOUSA1428104, HOUSA1441440 and HOUSA1491007) that were born in the 1960s [12].

**Table 1:** **The median CN of *HSFY *** **and *ZNF280BY *** **in 15 cattle breeds**

**Breed (full name)**	**Short name**	**Sample size (n)s**	**Median CN of *HSFY *** **(range)**	**Median CN of *ZNF280BY *** **(range)**	**Lineage**
Angus	ANG	14	186 (126–216)	257 (149–295)	*Bos taurus*
Beefmaster	BMA	14	168 (135–218)	259 (184–352)	*Composite*
Brahman	BRM	10	153 (108–265)	278 (152–337)	*Bos indicus*
Brown Swiss	BSW	14	204 (48–291)	247 (105–331)	*Bos taurus*
Charolais	CHL	10	239 (160–308)	300 (250–336)	*Bos taurus*
Gir	GIR	14	185 (122–207)	280 (244–341)	*Bos indicus*
Hereford	HFD	15*	202 (124–246)	229 (165–311)	*Bos taurus*
Holstein	HOS	257	204 (21–271)	226 (28–295)	*Bos taurus*
Jersey	JER	28	157 (122–219)	259 (171–351)	*Bos taurus*
Limousin	LMS	14	201 (57–238)	280 (47–370)	*Bos taurus*
Nelore	NEL	10	180 (85–209)	289 (171–326)	*Bos indicus*
Norwegian Red	NRC	9	209 (193–232)	211 (168–376)	*Bos taurus*
Qinchuan	QC	26	143 (102–197)	161 (111–269)	*Composite*
Red Angus	RGU	11	176 (110–247)	201 (88–356)	*Bos taurus*
Santa Gertrudis	SGT	14	175 (162–232)	296 (234–380)	*Composite*
Total		460	197 (21–308)	236 (28–380)	

*The bull used as the calibrator was not used for the median CN estimation for the Hereford breed.

### Primer design and CNV estimation

The sequences of *HSFY* (GenBank acc. no. NM_001077006) and *ZNF280BY* (GenBank acc. no. NM_001078120) were aligned to the bovine Y chromosome draft sequence assembly (GenBank acc. no. CM001061, Project ID: 20275) using Splign [26]. A total of 192 *HSFY* loci and 234 *ZNF280BY* loci were predicted/annotated from the draft assembly of the BTAY sequence (GenBank acc. no. CM001061) [2]. The paralogous sequences for *HSFY* and *ZNF280BY* were retrieved and aligned by MEGA 5.0 [27]. Based on these alignments PCR primers were designed from conserved regions using the Primer Premier 5.0 program (http://www.premierbiosoft.com/). The binding sites of each primer on the draft BTAY sequence and the predicted sizes of each amplicon based on an electronic PCR (http://www.ncbi.nlm.nih.gov/tools/epcr/) were listed in Additional file 1: Table S1 and Additional file 2: Table S2. A single-copy gene *DDX3Y* (*DEAD box polypeptide 3, Y-linked*, GenBank acc. no. NT182066) was used as a reference. The primer information and primer efficiencies were listed in the Table 2. Subsequently, the confirmation of the Y chromosome specificity of the designed primers, quantitative real-time PCR (qPCR) and equations used for determining CN of *HSFY* or *ZNF280BY* were followed exactly as described by Yue *et al.*[12], except that PCR annealing temperatures were 59°C and 64°C for *HSFY* and *ZNF280BY*, respectively. Primer efficiencies were measured according to the equation E = 10^[-1/slope]^ within every plate we run; and the slope was generated by a standard curve described earlier [12].

**Table 2:** The information of primers used in qPCR

**Primer name**	**Sequence**	**Annealing Tm**	**Primer efficiency**
HSFYF	5′-CTCCTAAGGATGAATCAACTG-3′	59°C	1.88
HSFYR	5′-CACAAGATCCTCAGACAAAGC-3′		
ZNF280BYF	5′-GAAATACCACACCACCTGCC-3′	64°C	1.88
ZNF280BYR	5′-GATCTGTAACTGCAAACCTGG-3′		
DDX3YF	5′ -ATCGTGGGCGGAATGAGTGT-3′	59°C or 64°C	1.95 at 59°C or 1.90 at 64°C
DDX3YR	5′ -CTTGGTGGAAGCGGTTTTGA-3′		

### Association and statistical analysis

In order to minimize technical error and to have an accurate CN estimation, raw qPCR data that showed a coefficient of variation (CV) > 1% between the duplicates were excluded from further analysis. The normality of the CN data was assessed with the Kolmogorov-Smirnov and Shapiro-Wilk normality tests [28,29]. Box plot analyses on the CN data were conducted to detect the outliers in all the breeds (populations) as a whole and in the Holstein population. Multiple pair-wise comparisons of median copy number (MCN) between breeds were analyzed using a nonparametric Mann-Whitney U test [30] with a Bonferroni correction [31]. In addition, the Mann-Whitney U test was also used to compare the MCN between groups that were classified based on the origin and formation of the cattle breeds, *i.e.* BTA, BIN, and composite (COM).

Initial analysis of the association between CN and reproductive traits were performed for Holstein bulls by a Pearson’s correlation test in SPSS 17.0 (SPSS Inc., IL, USA). A three-way Analysis of Variance (ANOVA) was applied to investigate the impact of the CN and the founder (of a paternal pedigree) on the SC, RLSC, PTM, IM, PNS and PIA using a general linear model in SPSS 17.0. Furthermore, a mix model of SAS 9.2 (SAS Institute Inc., Cary, NC, USA) was applied to investigate the impact of the CNV of Y-linked genes on RLSC, SC, PTM, IM, PNS and PIA, in which the sire was included as a random effect. A *P*-value ≤ 0.05 was considered statistically significant for each test.

## Results

### CNVs of *HSFY* and *ZNF280BY* across cattle breeds

**Figure 1:**
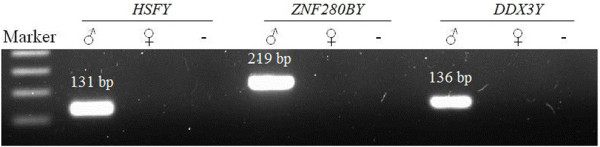
**Gel electrophesis of PCR products of the bovine *HSFY*, *ZNF280BY *and *DDX3Y*.** The primers of these three Y-linked genes amplified male specific bands with expected fragment size labeled above the band. Marker: 1 kb DNA ladder; ♂: the Hereford bull L1 Domino 99375 genomic DNA; ♀: female cattle genomic DNA; -: negative control (distilled water).

In order to validate the male specificity of primers used in this study, a routine PCR was run. The results demonstrated that every primer pair for the target genes, *HSFY* and *ZNF280BY*, or the single copy reference gene, *DDX3Y*, amplified a male-specific band with the expected fragment size, confirming that the designed primers are male-specific and can be used for the qPCR analysis in this study (Figure 1).

**Figure 2:**
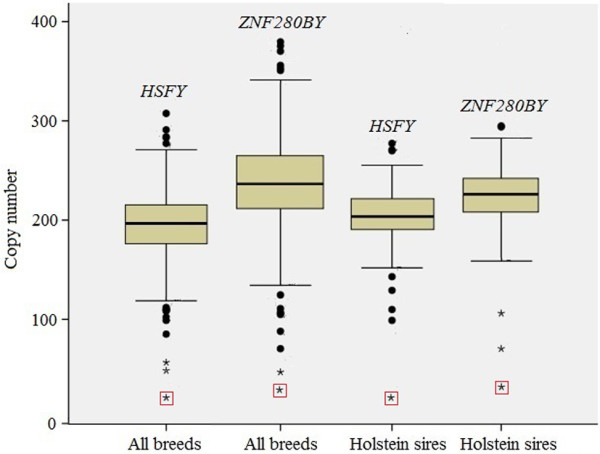
**Box plot analysis of the *HSFY *and *ZNF280BY *CN in cattle.** The outliers were indicated by a solid circle or an asterisk (extremely low CN), which include 12 bulls in all breeds and 9 bulls in Holstein for *HSFY*, while 14 and 6 for *ZNF280BY*, respectively. The animal that has the lowest CN of *HSFY* and *ZNF280BY* was marked with a red square.

**Figure 3:**
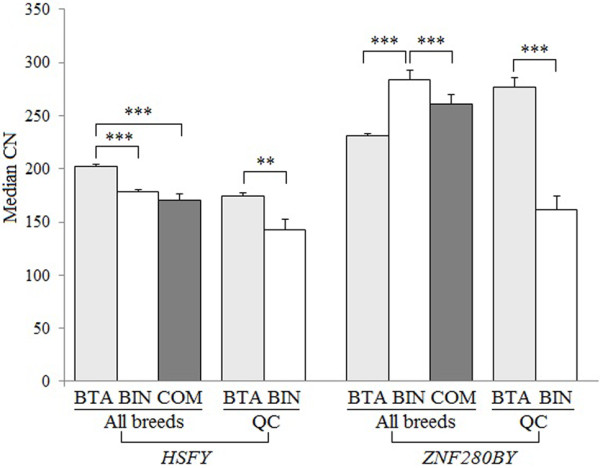
**Comparison of the CN of *HSFY *and *ZNF280BY *between the BTA and BIN lineages.** The CN of *HSFY* and *ZNF280BY* was significantly different between the lineages (*P* < 0.01).The MCN of *HSFY* in taurine cattle (BTA) was significant higher than that of indicine cattle (BIN) and composite cattle (COM). In contrast, the MCN of *ZNF280BY* in BIN was significant higher than that in BTA and composite cattle (COM). In the Qinchuan cattle, BTA-derived bulls had a significantly higher MCN of *HSFY* and *ZNF280BY* than BIN*-*derived bulls (*P* < 0.01). Y-axis represents the MCN of *HSFY* and *ZNF280BY*. The error bars represent standard errors. ** *P* < 0.01; *** *P* < 0.001.

The CN of *HSFY* in the calibrator was estimated to be 204, which is close to the 192 copies estimated from the draft sequence assembly of the very same Y chromosome [2].The MCN of *HSFY* was 197, ranging from 21 to 308 among 459 bulls tested (Figure 2). We found that the CN data did not fit the normal distribution (*P* < 0.05) in either the Holstein population that is the largest population (257 AI bulls) in the study or all populations of 15 breeds analyzed based on Kolmogorov-Smirnov and Shapiro-Wilk normality tests. Pair-wise comparisons of the MCN of *HSFY* between breeds were listed in Table 3, which indicated a significant difference among breeds (Table 3). Brahman bulls had the lowest MCN of *HSFY* (153, ranging 108-265), whereas Charolais bulls possessed the highest MCN (239, 106-308) (Table 1). When we analyzed the CN data based on the taurine (BTA) and indicine (BIN) lineages irrespective of the breed origin, we found that bulls in the BTA lineage had significantly higher MCN (202) than bulls in the BIN lineage (178) and the composite breeds (Beefmaster, Santa Gertrudis and Qinchuan, 170) (Figure 3).

**Table 3:** **Comparisons of the median CN of *HSFY *** **among 15 cattle breeds**

	**ANG**	**BMA**	**BRM**	**BSW**	**CHL**	**GIR**	**HFD**	**JER**	**LMS**	**NEL**	**NRC**	**QCT**	**RGU**	**SGT**	**HOS**
ANG	-														
BMA	nsd	-													
BRM	*	nsd	-												
BSW	nsd	nsd	*	-											
CHL	**	**	***	nsd	-										
GIR	nsd	nsd	**	nsd	**	-									
HFD	nsd	*	**	nsd	nsd	*	-								
JER	nsd	nsd	nsd	**	***	*	***	-							
LMS	nsd	*	**	nsd	nsd	nsd	nsd	**	-						
NEL	nsd	nsd	nsd	nsd	**	nsd	*	nsd	nsd	-					
NRC	*	**	**	nsd	nsd	***	nsd	***	nsd	**	-				
QCT	nsd	nsd	*	nsd	**	nsd	**	nsd	***	nsd	***	-			
RGU	nsd	nsd	nsd	nsd	*	nsd	nsd	nsd	nsd	nsd	*	nsd	-		
SGT	nsd	nsd	*	nsd	**	nsd	*	*	*	nsd	**	nsd	nsd	-	
HOS	**	***	***	nsd	*	***	nsd	***	nsd	**	nsd	***	**	***	-

Note: nsd: no significant difference; * *P* < 0.05; ** *P* < 0.01; *** *P* < 0.001. The full name of each breed is listed in Table 1.

The CN of *ZNF280BY* in the calibrator was estimated to be 239, almost identical to the CN (234) annotated from the Y chromosome sequence data [2]. The MCN was 236 with a range from 28 to 380 among the bulls tested. Like the *HSFY* gene family, the CN of *ZNF280BY* also did not fit the normal distribution in Holstein and other breeds with several bulls having CN in outliers (Figure 2); the MCN between breeds was significantly varied (Table 4); and Red Angus had the lowest MCN of 201, whereas Charolais bulls had the highest MCN of 300 (Table 1). However, unlike *HSFY*, *ZNF280BY* showed significantly higher MCN in the BIN lineage (284) than that in the BTA lineage (231), and the three composite cattle breeds had an intermediate MCN of 262 (Figure 3).

**Table 4:** **Comparisons of the median CN of *ZNF280BY *** **among 15 cattle breeds**

	**ANG**	**BMA**	**BRM**	**BSW**	**CHL**	**GIR**	**HFD**	**JER**	**LMS**	**NEL**	**NRC**	**QCI**	**RGU**	**SGT**	**HOS**
ANG	-														
BMA	nsd	-													
BRM	nsd	nsd	-												
BSW	nsd	nsd	nsd	-											
CHL	**	**	nsd	*	-										
GIR	*	**	nsd	nsd	nsd	-									
HFD	nsd	nsd	nsd	nsd	*	*	-								
JER	nsd	nsd	nsd	nsd	**	*	nsd	-							
LMS	nsd	nsd	nsd	nsd	nsd	nsd	nsd	nsd	-						
NEL	nsd	nsd	nsd	nsd	nsd	nsd	nsd	nsd	nsd	-					
NRC	nsd	nsd	nsd	nsd	**	**	nsd	nsd	*	nsd	-				
QC	nsd	*	nsd	nsd	nsd	nsd	*	nsd	nsd	nsd	**	-			
RGU	nsd	nsd	nsd	nsd	**	**	nsd	*	*	*	nsd	**	-		
SGT	**	***	nsd	*	nsd	nsd	**	**	nsd	nsd	**	nsd	***	-	
HOS	**	**	nsd	nsd	***	***	nsd	***	***	***	nsd	***	nsd	***	-

Note: nsd: no significant difference; * *P* < 0.05; ** *P* < 0.01; *** *P* < 0.001. The full name of each breed is listed in Table 1.

We analyzed a total of 26 bulls from a native Chinese cattle population of Qinchuan in the present study; 16 of which had a BTA Y chromosome, while the remaining 10 had a BIN Y [32]. We found that the BTA derived Y chromosome had a significant higher MCN of *HSFY* (174) and *ZNF280BY* (277) than the BIN derived Y chromosome (143 and 161) (Figure 3).

Box plot analyses of the CN data revealed that a small number of animals (~ 3%) were outliers who had significantly higher or lower CN (Figure 2). Of those, 7 and 4 outliers were identified for *HSFY* and *ZNF280BY* in the Holstein population, respectively (Figure 2). None of the outliers was part of the Holstein bulls with the SCR record. One of the outliers in Holstein had 21 copies of *HSFY* and 28 copies of *ZNF280BY* on its Y chromosome*,* the lowest copy number in both gene families among all individuals tested (highlighted in Figure 2).

### The correlation between the CN of *HSFY* and *ZNF280BY* on the bovine Y chromosome

To investigate the relationship of CN between *HSFY* and *ZNF280BY*, we included a third Y-linked gene family, *PRAMEY*, as a reference in our Pearson’s correlation analysis because *PRAMEY* is less amplified (compared to *HSFY* and *ZNF280BY*) on BTAY [2], and the CNV of *PRAMEY* was assessed with the same groups of animals in our previous study [12].We found that the CN of *ZNF280BY* was positively correlated to that of *HSFY* and *PRAMEY* (*P* < 0.001) in all bulls, irrespective of their origin. However, the CN of *HSFY* was not significantly correlated to the CN of *PRAMEY* (*P* = 0.637) (Table 5). When the cattle breeds were considered separately, the CN of these three genes was positively correlated to each other in only Holstein and Limousin (*P* < 0.0001), but not to other breeds (Table 5). The correlation coefficients were 0.488, 0.297, and 0.301 for *ZNF280BY-HSFY, ZNF280BY-PRAMEY,* and *HSFY-PRAMEY*, respectively, in the Holstein population, while 0.877, 0.862, and 0.886 in the Limousin population (Table 5). Furthermore, the CN of *ZNF280BY* was positively correlated to that of *HSFY* in Nelore and Qinchuan bulls, and that of *PRAMEY* in Brown Swiss and Gir bulls. In contrast, the CN of *HSFY* was negatively correlated to CN of *PRAMEY* in Jersey bulls. When the data were analyzed based on the BTA and BIN lineages, we observed that the CN of *ZNF280BY* was positively correlated with that of *HSFY* in both BTA (*P* < 0.0001), and BIN lineages (*P* = 0.006), while only positively correlated to that of *PRAMEY* in the BTA lineage (*P* < 0.0001) (Table 5).

**Table 5:** **The correlation between the CNV of *PRAMEY*, *HSFY *** **and *ZNF280BY***

**Groups/breeds**	***ZNF280BY*-*HSFY***		***ZNF280BY*-*PRAMEY***		***HSFY*-*PRAMEY***	
	**r**	***P***	**r**	***P***	**r**	***P***
Angus	0.385	0.273	0.094	0.825	0.484	0.111
Beefmaster	0.184	0.548	0.324	0.280	0.148	0.630
Brahman	0.284	0.458	0.078	0.855	-0.033	0.934
Brown Swiss	-0.215	0.526	0.739	0.015	-0.134	0.694
Charolais	0.223	0.565	-0.294	0.442	0.350	0.396
Gir	-0.031	0.921	0.997	0.003	0.283	0.717
Hereford	0.394	0.230	-0.222	0.511	0.185	0.610
Holstein	0.488	<0.0001	0.297	<0.0001	0.301	<0.0001
Jersey	0.278	0.249	-0.264	0.432	-0.728	0.017
Limousin	0.877	<0.0001	0.862	<0.0001	0.886	<0.0001
Nelore	0.858	<0.0001	-0.252	0.748	0.012	0.998
Norwegian Red	-0.320	0.485	-0.586	0.127	0.410	0.313
Qinchuan	0.755	<0.0001	-0.082	0.780	0.261	0.328
Red Angus	-0.469	0.172	0.302	0.468	-0.467	0.205
Santa Gertrudis	-0.239	0.432	0.327	0.527	0.101	0.829
*Bos taurus*	0.252	<0.0001	0.455	<0.0001	0.122	0.055
*Bos indicus*	0.478	0.006	0.081	0.766	0.008	0.977
All breeds	0.181	<0.001	0.443	<0.001	-0.026	0.637

### Association of the *HSFY* and *ZNF280BY* CNV with male reproductive traits in Holstein bulls

**Figure 4:**
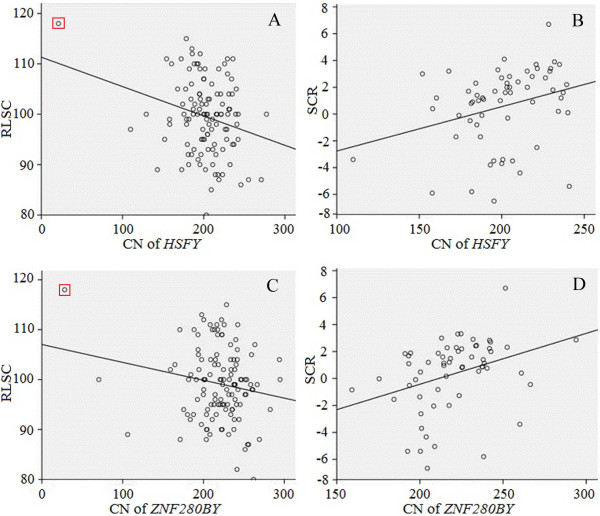
**Correlations between the CNV of *HSFY and ZNF280BY *and male reproductive traits in Holsteins.** The CNV of *HSFY* was **(A)** negatively associated with RLSC (r = -0.249, *P* = 0.008) and **(B)** positively associated with SCR (r = 294, *P* = 0.021). The *ZNF280BY* CNV was **(C)** negatively associated with RLSC (r = -0.174, *P* = 0.055) and **(D)** positively associated with SCR (r = 0.339, *P* = 0.008). Each circle represents one individual. The animal that has the lowest CN of *HSFY* and *ZNF280BY* was marked with a red square.

We performed the association analysis of CNVs of *HSFY* and *ZNF280BY* and their logarithm (base 10) transformation data with the three types of reproductive traits from 257 Holstein bulls, including testis size (RLSC and SC), semen quality (PTM, IM, PNS and PIA) and male fertility (SCR, NRR and RBE). The association analysis revealed that the RLSC was negatively associated with CNV (r = -0.249, *P =* 0.008) and Log_10_CNV of *HSFY* (r = -0.267, *P* = 0.004) (Table 6), and SC was negatively associated with Log_10_CNV of *HSFY* (r = -0.202, P = 0.025), while tended to be negatively associated with CNV of *HSFY* (r = -0.169, *P* = 0.073). For the *ZNF280BY*, RLSC is significantly associated with log_10_CNV of *ZNF280BY* (r = -0.239, *P* = 0.025), and tend to be associated with its CNV (r = -0.174, *P* = 0.055). SC was neither associated with CNV nor log_10_CNV of *ZNF280BY* (Table 6). These results suggested that a lower CN of *HSFY* and *ZNF280BY* is associated with a larger testis size (Figure 4). The results also demonstrated that the CNVs or Log_10_CNVs of *HSFY* and *ZNF280BY* were positively correlated to SCR, suggesting that a higher CN of *HSFY* and *ZNF280BY* is associated with a higher SCR (Figure 4). Furthermore, we investigated the relationship between reproductive traits and the sum of *HSFY* and *ZNF280BY* CNV (sCNV) and Log_10_sCNV. We found that sCNV and Log_10_sCNV of *HSFY* and *ZNF280BY* were negatively associated with RLSC, whereas positively associated with SCR. The remaining traits were not significantly associated with either sCNV or Log_10_sCNV (Table 6). Interestingly, the Holstein bull (mentioned above) who had the lowest copy number in both *HSFY* and *ZNF280BY* gene families had the largest RLSC among all animals tested, strongly supporting our association results that a lower CN of the two Y-linked genes is associated with a larger testis.

**Table 6:** **The Pearson’s correlation between the CNV of *HSFY *** **and *ZNF280BY *** **and the reproductive traits**

Traits*	***HSFY *** **CNV**		**Log**_**10**_**(*HSFY *** **CNV)**		***ZNF280BY *** **CNV**		**Log**_**10**_**(*ZNF280BY *** **CNV)**		**Sum CNV**		**Log**_**10**_**sCNV**	
	**r**	***P***	**r**	***P***	**r**	***P***	**r**	***P***	**r**	***P***	**r**	***P***
SC	-0.169	0.073	-0.190	0.044	-0.079	0.394	-0.113	0.218	-0.144	0.258	-0.158	0.117
RLSC	-0.249	0.008	-0.267	0.004	-0.174	0.055	-0.202	0.025	-0.218	0.030	-0.250	0.012
SCR	0.294	0.021	0.294	0.021	0.339	0.008	0.341	0.008	0.366	0.007	0.361	0.008
NRR	-0.005	0.961	-0.019	0.852	-0.162	0.106	-0.154	0.126	-0.118	0.267	-0.119	0.264
RBE	0.112	0.309	0.077	0.485	0141	0.210	0.075	0.508	0.164	0.162	0.096	0.414
PTM	-0.013	0.892	-0.031	0.748	0.045	0.632	0.002	0.981	-0.006	0.958	-0.024	0.818
IM	-0.061	0.533	-0.045	0.647	-0.087	0.359	-0.082	0.388	-0.115	0.272	-0.079	0.450
PNS	0.144	0.243	0.058	0.550	0.016	0.863	-0.004	0.969	0.048	0.647	0.017	0.874
PIA	-0.019	0.846	-0.002	0.986	-0.014	0.866	-0.007	0.940	-0.046	0.667	-0.014	0.895

*See full name in the Methods section.

In order to check whether the outlier bulls identified in the box plot analysis have any significant effect on the association analyses, we re-calculated the Pearson’s correlation coefficient by excluding the outliers from the data set. The results indicated that these outliers had considerable effect on the association results. Taken away the outliers from the analysis led to a non-significant association between testis size and CNVs of *ZNF280BY* and *HSFY* (*P >* 0.05, Table 7).

**Table 7:** **The Pearson’s correlation between the CNV of *HSFY *** **and *ZNF280BY *** **and the reproductive traits after excluding the outliers from the data set**

Traits*	***HSFY *** **CNV**		***ZNF280BY *** **CNV**	
	**r**	***P***	**r**	***P***
SC	-0.085	0.187	-0.079	0.197
RLSC	-0.150	0.057	-0.131	0.078
NRR	-0.037	0.714	-0.153	0.131
RBE	0.126	0.261	0.182	0.111
PTM	-0.064	0.527	0.122	0.206
IM	-0.011	0.910	-0.005	0.958
PNS	0.119	0.239	0.097	0.314
PIA	-0.018	0.858	0.044	0.654

*See full name in the Methods section.

To further investigate the effect of low and high CN of *HSFY* and *ZNF280BY* on the bull performance, we simply grouped the140 Holstein bulls (whose phenotypic data were available for SC, RLSC, PTM, IM, PNS and PIA) into two groups based on a cut-off threshold of MCN for *ZNF280BY* (223) and *HSFY* (203) in these 140 bulls (Table 8). Statistical analysis indicated that the difference between the low and high groups of the *HSFY* CN was significant in RLSC (*P* = 0.008) and closed to be significant in SC (*P* = 0.063), but not significant in the remaining four traits (PTM, IM, PNS and PIA) (*P* > 0.05) (Table 7). For the *ZNF280BY*, there is no significant difference between the low and high CN groups (Table 8). These results, from a different angle, confirmed that bulls with a low CN of *HSFY* tend to have a large testis size.

**Table 8:** **Reproductive performance of Holstein bulls with a high and low MCN of *HSFY *** **and *ZNF280BY***

Reproductive traits*	***HSFY***			***ZNF280BY***		
	**Low MCN**	**High MCN**	***P *** **value**	**Low MCN**	**High MCN**	***P *** **value**
	**(CN < 203, n = 56)**	**(CN ≥ 203, n = 58)**		**(CN < 223, n = 60)**	**(CN ≥ 223, n = 62)**	
SC (cm)	40.72 ± 0.44	39.62 ± 0.38	0.063	40.11 ± 0.43	39.81 ± 0.39	0.616
RLSC (%)	101.41 ± 0.99	97.74 ± 0.90	0.008	100.11 ± 0.97	98.24 ± 0.89	0.157
PTM (%)	77.59 ± 0.28	77.62 ± 0.41	0.962	77.51 ± 0.29	77.88 ± 0.38	0.444
IM (%)	34.37 ± 0.27	34.05 ± 0.46	0.540	34.22 ± 0.30	34.15 ± 0.42	0.900
PNS (%)	77.92 ± 1.09	78.38 ± 1.04	0.762	77.18 ± 1.00	79.52 ± 1.02	0.106
PIA (%)	80.34 ± 0.39	80.16 ± 0.44	0.440	80.24 ± 0.36	80.31 ± 0.42	0.891

*See full name in the Methods section.

### Relationship between bull pedigrees and their *HSFY* and *ZNF280BY* CNVs and reproductive traits in Holstein

Among the 257 Holstein bulls investigated, 192 had paternal pedigree information [12]. These bulls were descendants of only 4 patrilineal founders (HOUSA1427381, HOUSA1428104, HOUSA1441440 and HOUSA1491007) that were born in the 1960s. The MCN of *HSFY* and *ZNF280BY* for each founder lineage was re-calculated (Additional file 3: Figure S1). The MCN of *HSFY* among the descendants was 203,198 and 205, for the founder HOUSA1427381, HOUSA1441440 and HOUSA1491007, respectively (the fourth founder had only one descendant in the tested populations), while the corresponding MCN of *ZNF280BY* for these founders was 221, 220 and 223. The MCN variations were not significant between these founder lineages (*P* > 0.05).

To investigate whether the founder effect has any impact on our gene CNV and male reproductive trait association analysis, we performed three-way ANOVA within the two relative large descendant population HOUSA1427381 (40 individuals) and HOUSA1491007(81 individuals). Our results (Table 9) revealed that the founders had no significant effects on the reproductive traits, and the interactions between the founders and the CNVs of the Y-linked genes had no significant effects either.

**Table 9:** **The *P *** **values of the three-way ANOVA**

Reproductive traits*	**Founder**	***HSFY***	***ZNF280BY***	***HSFY *** **× Founder**	***ZNF280BY *** **× Founders**	***ZNF280BY *** **× *HSFY***	**Founder × *HSFY *** **× *ZNF280BY***
SC	0.428	0.373	0.094	0.493	0.363	0.401	0.485
RLSC	0.270	0.691	0.490	0.580	0.075	0.152	0.926
PTM	0.500	0.245	0.578	0.279	0.619	0.346	0.326
IM	0.438	0.822	0.673	0.971	0.963	0.190	0.721
PNS	0.673	0.836	0.922	0.057	0.829	0.130	0.955
PIA	0.502	0.858	0.779	0.367	0.255	0.165	0.294

*See full name in the Methods section.

Out of the 192 bulls, 94 were half-sib brothers from 27 sires. In order to exclude the sire effect on the above CNV and testis size association analyses, we included sire in the mix model as a random effect (see Methods). We found that the CNV of *HSFY* had a significant effect on SC (*P* = 0.022) and RLSC (*P* = 0.002), whereas the CNV of *ZNF280BY* had no significant on testis size (*P* = 0.09 on RLSC and *P* = 0.511 on SC).

## Discussion

### The CN of the *HSFY* and *ZNF280BY* gene families are variable across cattle breeds

In this study, we investigated the CN of two Y-linked gene families, *HSFY* and *ZNF280BY,* among 15 cattle breeds by a modified qPCR method [12] that has some advantages over the previously described method [16]. We estimated that 204 and 239 copies of *HSFY* and *ZNF280BY* are present on the Y chromosome of a Hereford bull (L1 Domino 99375), the calibrator in this study, whose DNA was used for the bovine Y sequence project (NCBI Project ID: 20275). These results were in close agreement with the 192 and 234 copies (of *HSFY* and *ZNF280BY)* annotated from the draft assembly of the BTAY sequence (GenBank acc. no. CM001061), indicating the reliability of our qPCR method. For the first time, we were able to show that the Y-linked *HSFY* and *ZNF280BY* gene families, like the *TSPY* family, were extensively amplified on the bovine Y chromosome. The CN varies significantly from 21 to 308 for *HSFY*, and 28 to 380 for *ZNF280BY* among all bulls, with a variable MCN among 15 breeds tested. We noticed that the estimation of 197 copies (MCN) for the bovine *HSFY* in this work was significantly higher than the ~70 copies reported by Hamilton *et al*. [17]. Despite the possible influence from the cattle breeds as the two studies used different number of bulls/breeds, we believe that the most important reason for the *HSFY* CN discrepancy between the two studies is the *HSFY* gene PCR primer design as the draft BTAY sequence was not available when Hamilton *et al.* carried out their study. After carefully aligning the PCR primer sequences against the draft assembly of the BTAY sequence (GenBank acc. no. CM001061), we understood that the *HSFY* primer pairs designed by Hamilton *et al*. matched approximately 120 loci (with a 100% identity), suggesting that their primer pairs targeted ~ 60% of all *HSFY* loci on the sequenced Y chromosome. Further, our data did not support the previous report that found no CNV of *HSFY* among 24 randomly selected Holstein bulls [17]. In contrast, we found that the CNV of *HSFY* was not only present, but also with the largest variation range (21-271, Table 1) in the Holstein population. In addition to genetic factors that may determine the variation, the largest CNV range of *HSFY* observed in this group of Holsteins was most likely due to a much larger number of Holstein bulls tested in the present study.

We further analyzed the CNV among three patrilineal lineages in the Holstein population and found considerable variations among the Y chromosomes within any given Y-lineage even though the median CN was not significantly different among these three Y-lineages. These results were expected as both *HSFY* and *ZNF280BY* gene families are located in the palindrome-like repeats (see discussion below) [2,3], which provide a genetic mechanism (*i.e.* intrachromosomal recombination between repeated homologous sequences) for deletion (or amplification) of the Y-linked genes (or DNA segments). We believe that our current observation in the bovine Y CNV is similar to the earlier reports on the human Y chromosome P1 (Palindrome 1) region where the AZFc (Azoospermia Factor c) is located [6,33,34]. AZFc is the most common known genetic cause of severe spermatogenic failure in men [34]. Frequent deletions in this region have been identified in patients with azoo and/or oligospermia and account for ~ 15% of all infertile cases [33]. Within a human Y-lineage, *de novo* deletion in the AZFc region was estimated at a rate of 1.1 × 10^-5^ to 1.4 × 10^-4^ per father-to-son transmission of the Y chromosome [34]. As a consequence of lack of recombination during meiosis in the MSY region, these *de novo* AZFc-related deletions will be kept in the son’s Y-lineage. Although there is no detailed research on the rate of the bovine Y mutation (deletion, insertion, etc.), results from the human Y chromosome provide a reference for the future research in cattle. It is worth noting that the Holstein bull that had the lowest CN of both *HSFY* and *ZNF280BY* on its Y chromosome had the largest relative testis size (RLSC) among all bulls tested (Figure 4A and C). It is likely that this particular Y chromosome has a large deletion in the *HSFY*- and *ZNF280BY*- related region, which, from a different angle, supports our finding that less CN is associated with larger testis in Holsteins.

### The amplification of the *HSFY* and *ZNF280BY* gene families are different between the taurine and indicine Y chromosomes

The previous studies indicated that bovine Y ampliconic region consists of ∼ 80 palindrome-like repeat units (∼ 420 kb per unit) and that each unit contains one to three copies of the corresponding gene families (*TSPY*, *HSFY*, *ZNF280BY* and *ZNF280AY*) [2,3]. The *TSPY* gene family has been evidenced to have undergone extensive amplification (50-200 copies) across the entire ampliconic region (~ 30 Mb) during evolution [2,16]. The CNV of *HSFY* and *ZNF280BY* observed in current study further confirmed the extensive amplification of the *HSFY* and *ZNF280BY* on BTAY. In addition, we found that CN between *HSFY* and *ZNF280BY* was positively correlated (Table 5), which was accordant with previous finding that *HSFY* and *ZNF280BY* were amplified together on BTAY [2].

It was interesting to see that the CN between *PRAMEY* and *ZNF280BY* was only positively correlated in the BTA lineage, but not in BIN lineage. Recent studies demonstrated that *PRAMEY* and *ZNF280BY*, unlike the *TSPY* and *HSFY* that are present in other mammalian Y chromosomes, were bovid-specific and were derived from the transposition of the *ZNF280B/ZNF280A/PRAME* block on BTA17 and amplified separately thereafter on BTAY [3,4]. The positive correlation of CN between *PRAMEY* and *ZNF280BY* observed in this work further supports the previous findings [3]. Our data on *HSFY* revealed that bulls in the BTA lineage had a significantly higher MCN of *HSFY* than bulls in the BIN lineage (202 copies vs. 178 copies). In contrast, bulls in the BTA lineage had a significantly lower MCN of *ZNF280BY* than those bulls in the BIN lineage (231 vs. 284). Though the mechanism behind variations of gene CN is still largely unknown, it is believed that gene CN may originally depend on functional requirement and natural selection [35]. If so, *HSFY* could be one of the examples for functional selection. As its name indicated, *HSFY* may play an important role in heat reaction for zebu to adapt to the high temperatures in the tropical countries by reducing the CN of *HSFY*.

As a consequence of the bovid evolution, the only visible morphological difference identified between BTA and BIN genome at a cytogenetic level is the Y chromosome. The BTA Y chromosome is metacentric, while the BIN Y is acrocentric [36]. This morphological difference is believed to be caused by a Y chromosome rearrangement [37]. Obviously, the difference observed in the CN correlation of the three Y-linked gene families (*PRAMEY*, *HSFY* and *ZNF280BY*) between BTA and BIN lineages in this study (Table 5) is related to the origin of the BTA and BIN Y chromosomes. Since the current researches are focused mainly on the BTA Y chromosome, including the BTAY sequencing project, the data from this study clearly indicate that more research is needed on the BIN Y lineage in order to understand the mechanism underlying the Y-linked gene expansion and their function in male reproduction.

### The CNVs of Y-linked gene families could be used as potential DNA markers for male fertility selection

The majority of the genes on the Y chromosome is found to be involved in male spermatogenesis and, hence, are closely associated with male fertility [1]. Previous studies have shown that CNV of *TSPY* was associated with male fertility in human and cattle [9]– [11]. The CNV of a bovid-specific Y-linked gene, *PRAMEY*, was found to be negatively associated with testis size, PNS and NRR in cattle [12]. Concerning the relationship of CNVs and reproductive traits in this study, the CNVs of *HSFY* and *ZNF280BY* are negatively correlated with testis size (SC and RLSC), but positively associated with SCR. SCR is a newly developed method to evaluate the fertility of bull in the US, and includes several components in the evaluation model, *i.e.* the expanded service sire term, inbreeding of the bull, inbreeding of the embryo from the mating, age of the bull, AI organization combined with year of the mating, and fertility of the cow a bull is mated with (http://aipl.arsusda.gov/reference/arr-scr1.htm). In contrast, SC and RLSC are simply and directly quantitative traits. In a natural mating system, SC is important for fertility (more sperm with larger testicles) [38]. This may not be true in AI because semen is diluted to allow many breeding from a single ejaculate. Thus, pregnancy rates (like SCR) achieved by different sires are minimized [39]. Therefore, it is not surprising to observe this conflict tendency (positive vs. negative) in association analysis.

The molecular mechanism by which a CNV can affect transcription, translation, or even a phenotype is largely unknown. Previous studies revealed that CNVs can be inversely correlated to their mRNA expression [11,40,41]. However, how the CNV of *ZNF280BY* or *HSFY* impacts the transcription and translation of the gene family has not been studied yet. The human Y chromosome comprises 2 copies of *HSFY*. A deletion on one or both copies can lead to severe impacts on fertility [18,20]. In contrast, cattle have about 200 copies of *HSFY* and *ZNF280BY* which may allow for many “backups” in case one or more of the copies undergo mutations, thereby minimizing/eliminating harmful effects on fertility as suggested by Hamilton *et al.*[17].

Testicular size is a very unique trait for a bull and has been used as a predictive indicator for output of sperm cells for yearling bulls [42]. However, very few genetic markers have been identified to date for a selection purpose due to the lack of molecular genetic study in bull fertility. The CNVs of *HSFY* and *ZNF280BY* found in this study, and that of *PRAMEY* found in our previous study [12], were all negatively associated with testis size. These results indicated that the CN of these Y-linked genes may provide a new insight for testis selection in an early age in cattle.

## Conclusion

We confirmed here that the two Y-linked gene families, *HSFY and ZNF280BY*, were extensively and differentially expanded on the BTA and BIN Y chromosomes. The copy number of these gene families is highly variable among individuals and breeds. The CNVs of *HSFY* and *ZNF280BY* are negatively associated with testis size, which may serve as valuable markers for male fertility selection in an early age.

## Competing interests

The authors declare that they have no competing interests.

## Authors’ contributions

WSL and XPY conceived the experiments; XPY carried out the experiments; XPY, CD and TCC analyzed the data; XPY and WSL wrote the manuscript. JMD and CEM collected the semen samples and the phenotypic data. TCC, CZL, JMD and CEM revised the manuscript. All co-authors read and approved the final manuscript.

## Supplementary Material

Additional file 1: Table S1 The binding sites of the *HSFY primers* against the bovine Y chromosome draft sequence assembly. Click here for file

Additional file 2: Table S2 The binding sites of the *ZNF280BY primers* against the bovine Y chromosome draft sequence assembly. Click here for file

Additional file 3: Figure S1 The pedigree information of 140 Holstein bulls whose phenotypic data were available for this study. Click here for file
