# Supplementary figures and images for: Copy number variations of the extensively amplified Y-linked genes, HSFY and ZNF280BY, in cattle and their association with male reproductive traits in Holstein bulls
Source: BMC Genomics. 2014 Feb 8;15:113. doi: 10.1186/1471-2164-15-113 (PMC3924399; doi:10.1186/1471-2164-15-113)

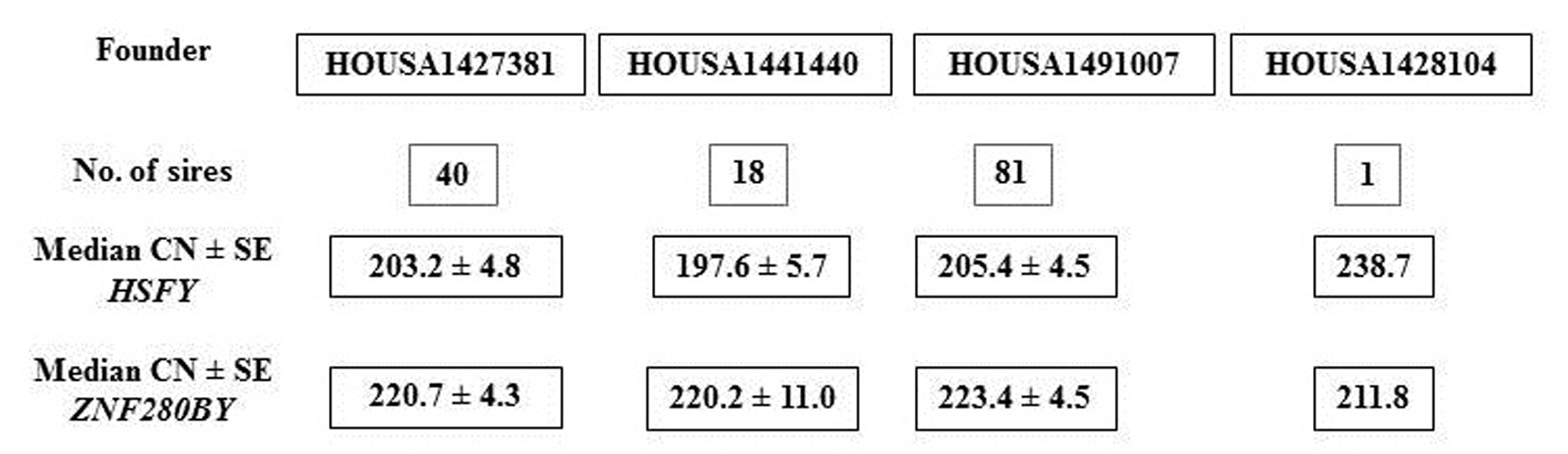

Supplement: Additional file 3: Figure S1 — The pedigree information of 140 Holstein bulls whose phenotypic data were available for this study. [file 1471-2164-15-113-S3.tiff]
